# Online marketing practices of regenerative medicine clinics in US-Mexico border region: a web surveillance study

**DOI:** 10.1186/s13287-021-02254-4

**Published:** 2021-03-18

**Authors:** Javier Chavez, Neal A. Shah, Severin Ruoss, Raphael E. Cuomo, Samuel R. Ward, Tim K. Mackey

**Affiliations:** 1 Masters Program in Clinical Research, UC San Diego – School of Medicine, San Diego, CA USA; 2 Department of Healthcare Research and Policy, UC San Diego – Extension, 8950 Villa La Jolla Drive Suite A124, San Diego, CA 92037 USA; 3 Global Health Policy and Data Institute, San Diego, CA USA; 4 Department of Orthopaedic Surgery, UC San Diego – School of Medicine, San Diego, CA USA; 5 Department of Anesthesiology and Division of Global Public Health, University of California, San Diego School of Medicine, San Diego, CA USA; 6 Department of Orthopaedic Surgery and Department of Radiology, UC San Diego – School of Medicine, San Diego, CA USA; 7 S-3 Research, LLC, San Diego, CA USA

**Keywords:** Stem cell therapy, Regenerative medicine, Online marketing, Mexico, Regulatory science

## Abstract

**Introduction:**

The potential of regenerative medicine to improve human health has led to the rapid expansion of stem cell clinics throughout the world with varying levels of regulation and oversight. This has led to a market ripe for stem cell tourism, with Tijuana, Mexico, as a major destination. In this study, we characterize the online marketing, intervention details, pricing of services, and assess potential safety risks through web surveillance of regenerative medicine clinics marketing services in Tijuana.

**Methods:**

We conducted structured online search queries from March to April 2019 using 296 search terms in English and Spanish on two search engines (Google and Bing) to identify websites engaged in direct-to-consumer advertising of regenerative medicine services. We performed content analysis to characterize three categories of interest: online presence, tokens of scientific legitimacy, and intervention details.

**Results:**

Our structured online searches resulted in 110 unique websites located in Tijuana corresponding to 76 confirmed locations. These clinics’ online presence consisted of direct-to-consumer advertising mainly through a dedicated website (94.5%) or Facebook page (65.5%). The vast majority of these websites (99.1%) did not mention any affiliation to an academic institutions or other overt tokens of scientific legitimacy. Most clinics claimed autologous tissue was the source of treatments (67.3%) and generally did not specify route of administration. Additionally, of the Tijuana clinics identified, 13 claimed licensing, though only 1 matched with available licensing information.

**Conclusions:**

Regenerative medicine clinics in Tijuana have a significant online presence using direct-to-consumer advertising to attract stem-cell tourism clientele in a bustling border region between Mexico and the USA. This study adds to existing literature evidencing the unregulated nature of online stem cell offerings and provides further evidence of the need for regulatory harmonization, particularly to address stem cell services being offered online across borders.

**Supplementary Information:**

The online version contains supplementary material available at 10.1186/s13287-021-02254-4.

## Background

Regenerative medicine has undergone significant advances in the past few decades, positioning it to become a critical tool in the future of modern medicine. Incorporating several disciplines, from stem cell and molecular biology to bioengineering, the field of stem cell-based interventions (SCBIs) tackles issues that deal with healing, regeneration, or replacement of cells and tissues [1]. Preclinical data and some early phase studies have shown promise in applications for diverse fields, ranging from orthopedic surgery to oncology, cardiology, and neurology [2]. Yet, the full spectrum of its scientific and clinical applications remains to be seen.

Since its inception, however, regenerative medicine and stem cell therapies have generated a great deal of conversation, skepticism, and debate about its implementation as a form of clinical treatment among scientists, clinicians, lay press, prospective patients, and regulatory agencies [3–5]. Hence, the great potential stem cell therapies have shown in pre-clinical studies is also associated with specific barriers limiting clinical translation, including the need to generate more evidence of efficacy and safety, concerns about unregulated and unsubstantiated marketing prior to treatments being approved or adequately tested, and legal and regulatory challenges associated with authorization and use [6].

In particular, regulatory aspects of stem cell treatments have been a point of contention, with the need to develop and mature an appropriate regulatory framework specific to regenerative medicine interventions proving difficult for many countries [7–10]. For example, on November 2017, the US Food and Drug Administration (FDA) published two guidance documents to clarify parts of Title 21 of the Code of Federal Regulations part 1271, which deals with human cells, tissues and cellular and tissue-based products (HCT/P’s) [11, 12]. These were needed due to the fact that some clinics were exploiting the vague definitions of “minimal manipulation” or “homologous use” in the context of classifying their products as “351” or “361” products under the Public Health Services Act (PHS Act) [13].

Despite actions by the FDA, the USA and other countries have not yet adopted comprehensive regulatory harmonization approaches on the matter, including in the context of addressing potential conflict between what is allowed within the scope of the practice of medicine and formal regenerative medicine product approval processes [6]. Such is also the case in Mexico, a stem cell tourism destination; the regulatory agency “Comisión Federal para la Protección contra Riesgos Sanitarios” (COFEPRIS) has regulations in place for stem cell collection facilities and biobanks, but there are no specific regulations for the applications of these therapies [14]. Despite proposals and requests for regulating these therapies since 2015 by the medical community, including a statement by the Mexican National Academy of Medicine as recently as 2018 expressing concern for the lack of regulations on SCBI’s, no official regulatory framework, “Norma Official Mexicana” (NOM), has been approved as of this date [14, 15].

Due to less stringent regulations and lower costs, Mexico has become a home for medical tourism, including for regenerative medicine and stem cell therapies. Specifically, the city of Tijuana has been identified as one of the most prominent locations for medical tourism in Mexico due to its close proximity to the USA via the San Diego/Tijuana border [14, 15]. The unique phenomenon of this regional stem cell tourism industry is due in part to this proximity, but also the aforementioned lack of stringent stem cell therapy regulations by COFEPRIS compared with the FDA, giving rise to more experimental therapies and greater access to forms of treatment. Given these issues, Tijuana is an important region to further examine in the context of regenerative medicine services marketed online that can easily cross national borders of close proximity.

Previous studies examining online marketing of stem cell services have used generalized searches, focusing on characterizing either the global or national/country-level characteristics of stem cell online offerings, but did not look at city-level data with their searches, and were only conducted in the English language [16–20]. In order to better elucidate the specific characteristics of stem cell tourism in this popular cross-border region, we conducted an observational, cross-sectional study to identify Tijuana-based regenerative medicine clinic websites using online direct-to-consumer advertising to market to prospective clientele. Specifically, we examined the number of businesses, diversity of regenerative medicine/stem cell-based interventions offered, and pricing reported by websites of clinics located in Tijuana.

This Internet surveillance study explored the Tijuana area specifically by incorporating both English and Spanish languages in structured search engine queries to assess both the domestic and medical tourism market for stem cell treatments offered. We also conducted a sub-analysis of regenerative medicine clinics located in San Diego, including those near the San Diego/Tijuana border, for the purposes of providing an objective comparison of what services are marketed in these different jurisdictions of close proximity. The objective of the study was to identify and characterize regenerative medicine marketing in the US-Mexico border region in order to assess potential patient safety risks and needed regulatory responses.

## Methods

### Search terms and strategy

To simulate what a potential patient or consumer interested in regenerative medicine/stem cell therapy services might be exposed to online, we conducted structured online search queries from March to April 2019, using *Google* and *Bing* search engines as these platforms are cited as the #1 and #2 most popular search engines globally. Search terms used for this study were derived from a combination of keywords that targeted geographic study locations of interest (Tijuana and San Diego), specific cell and treatment types (e.g., SCBI and platelet-rich-plasma [“PRP”]), as well as some common conditions purportedly treated by stem cell therapies as detected in prior studies using online search methodologies [16, 17]. Search terms were originally identified in English and then translated to Spanish resulting in a bilingual search mirrored on both search engines. The total number of search terms used in this study was 296 (148 in each language) (see Supplementary Table 1 for full list of search terms used).

For our web-browser settings, we used Google Chrome with all personal accounts signed out and set to “incognito” mode and Bing in the InPrivate mode to minimize the influence of user data, such as browsing history, cookies, and search history, on study results. This mode, however, still retains information about the geographic location linked to the IP address of the computer that is used to conduct searches. To account for the potential influence of the IP address on search results, we conducted structured web queries in physical locations in both San Diego, USA, and Tijuana, Mexico, with corresponding IP addresses originating in both countries. Based on sampling methodologies of other published studies, we reviewed the first five pages of organic search results.

All structured search results were queried and extracted with all website/hyperlink duplicates removed. Websites were then manually annotated if they met all of the following inclusion criteria: (1) operating a website or Internet/social media account with an online presence (e.g., clinic website, Facebook page, YouTube channel, etc.), (2) actively marketing putative regenerative medicine (e.g., PRP and SCBIs) on humans), and (3) reporting physical business locations of clinics within Tijuana or San Diego. Search results were excluded if any of the following conditions were met: (a) determined to be a website promoting a clinic or business located outside of the targeted geographical area; (b) the website was not operational; (c) the website or other online sources indicated the business or clinic had closed permanently; (d) offered regenerative medicine or SCBI’s solely marketed for veterinary use; (e) the businesses provided products by mail only; (f) the website marketed equipment or supplies for regenerative medicine/SCBI, but did not provide therapy; or (g) websites promoted a network of businesses but did not themselves provide therapy.

### Content analysis

After identifying websites per our structured online search query protocol, we conducted content analysis of the websites that met our inclusion criteria. Sixty-three website characteristics were coded into variables of interest. These variables are categorized into three major thematic groups of interest: (1) online communication and partnership profile, (2) claims to scientific legitimacy, and (3) intervention details. The first category “online communication and partnership profile” captures data that describes how active clinics are in their online marketing and how open to communication they are with prospective patients or other professionals. With this publicly available data we were able to produce a map of all the unique physical addresses corresponding to clinics detected in this study. The second category “claims to scientific legitimacy” captures data that businesses could use as tokens of scientific legitimacy for marketing their services. Because it is difficult to determine scientific legitimacy without having access to specific protocols, we focused on data features such as affiliations, claimed licenses or approvals from government or regulatory agencies, and self-published information (such as blog posts) or claimed evidence supporting services (from third party sources; peer reviewed or lay press). Finally, “intervention details” were captured, from cost, to cell types and sources as well as route of administration. Special care was taken to highlight variables relating to risk communication, therapy outcome expectations, and offering follow-up visits, as they are all important data points that reflect the types of stem cell therapies offered and the levels of transparency associated with clinical practices.

A complete description of the variables collected as part of this study is provided in Supplementary Table 2.

### Statistical analysis

We conducted statistical analysis using variables generated in the website content analysis to describe and compare characteristics of regenerative medicine clinics reviewed that had business addresses in Tijuana and for our sub-analysis that included a review of stem cell clinics marketing services online that were located in San Diego. Descriptive statistics were computed to relay information about website characteristics for clinics in Tijuana and San Diego. To compare characteristics of clinics in Tijuana with those for clinics in San Diego, analysis was conducted with inferential statistics calculated. For continuous variables, we reported means and assessed differences with independent sample *t* tests. For categorical and binary variables, we reported proportions and assessed differences using Pearson chi-square tests. Statistical analysis was conducted using SPSS version 26 (IBM: Armonk, NY).

## Results

**Fig. 1:**
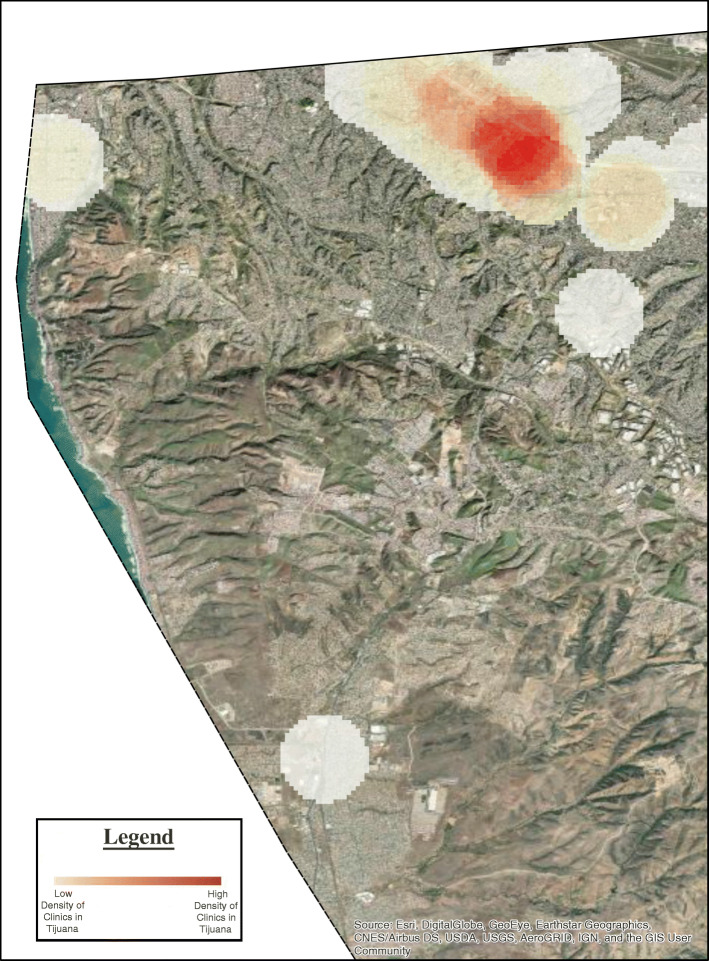
Density plot map of regenerative medicine businesses/clinics in Tijuana, Mexico, reviewed in this study

Our structured web searches consisted of 296 English and Spanish language search terms conducted on two different search engines from physical locations with two different IP addresses, one in Tijuana and the other in San Diego. In total, 1184 distinct web searches were conducted, and 5920 pages of results were produced and reviewed. After applying our inclusion and exclusion criteria and removing duplicates, we produced a final list of 257 websites including 110 detected in Tijuana, Mexico. Of the 110 websites, 76 were confirmed as unique stem cell/regenerative medicine clinics located in Tijuana (see Table 1). We found that there was geographic clustering in Tijuana along an area known as “Zona Rio,” which is a commercial zone that harbors many offices and businesses, including medical offices and which is widely known as a medical tourism destination (Fig. 1). Below we provide a breakdown of our content analysis based on website characteristics of interest.

**Table 1:** Output of metrics describing characteristics of websites for regenerative medicine clinics in Tijuana and San Diego

Category	Characteristic	San Diego metrics	Tijuana metrics
Online Communication and Partnership Profile	Facebook presence	76.9%	65.5%
	Twitter presence	43.5%	26.4%
	Other online presence	63.9%	37.3%
	Address provided	97.3%	88.2%
	Phone number provided	99.3%	97.3%
	Email provided	63.9%	68.2%
	Contact form	95.9%	85.5%
	Multiple clinics in area	27.9%	6.4%
	Partners mentioned	2.0%	12.7%
	Links to suppliers or other clinics	3.4%	10.9%
	Language: English	95.2%	40.0%
	Language: Spanish	0.0%	40.0%
	Language: Both English and Spanish	4.8%	20.0%
	Partner location: Mexico	0.0%	78.6%
	Partner location: USA	100%	0.0%
	Partner location: Mexico and USA	0.0%	1.0%
	Partner location: Other	0.0%	2.0%
Claims to Scientific Legitimacy	Affiliation with academia	7.5%	0.9%
	Affiliation with hospital/medical center	12.9%	13.6%
	Affiliation with society/network	27.9%	13.6%
	Affiliation with other authority	2.0%	6.4%
	Clinical trials conducted	5.4%	5.5%
	License for regenerative medicine	0.0%	11.8%
	Accreditation from professional org.	25.9%	20.9%
	FDA Approval	7.5%	1.8%
	COFEPRIS Approval	0.0%	12.7%
	Patent pending	0.0%	0.0%
	Patent approved	0.7%	0.9%
	Evidence cited: any (including testimonials)	39.5%	33.6%
	Evidence cited: peer-reviewed	9.5%	5.5%
	Evidence cited: lay press/blogs	4.8%	3.6%
	Evidence cited: self-publication	17.7%	6.4%
Intervention Details	Number of diseases treated (mean)	6.04	8.15
	Cost per Session	$1523.04	$3691
	PRP Offered	87.1%	67.3%
	Follow-up Mentioned	6.1%	11.8%
	Expectation: Unclear	17%	49.1%
	Expectation: Improvement	76.9%	48.2%
	Expectation: Cure	0.7%	0.0%
	Risk: Not mentioned	41.5%	55.5%
	Risk: Safe	51.0%	38.2%
	Risk: Minor	5.4%	6.0%
	Risk: Moderate	2.0%	0.0%
	Risk: Major	0.0%	0.0%
	Tissue origin: USA	84.4%	0.9%
	Tissue origin: Mexico	0.0%	60.0%
	Tissue origin: Unspecified	15.6%	39.1%
	Tissue source: Autologous	84.4%	61.8%
	Tissue source: Allogenic	13.6%	20.0%
	Tissue source: Xenogenic	1.4%	1.8%
	Tissue source: Unspecified	10.9%	32.7%
	Cell type: Adult Stem Cells	21.8/%	29.1%
	Cell type: Embryonic Stem Cells	0.0%	3.6%
	Cell type: IPSC	0.0%	0.0%
	Cell type: Cord, amniotic, or placental	13.6%	8.2%
	Cell type: Unspecified	11.6%	26.4%
	Route: Intrathecal	0.7%	8.2%
	Route: Oral	0.0%	0.0%
	Route: Topical	0.7%	0.9%
	Route: Intravenous	6.8%	14.5%
	Route: Intramuscular	0.7%	4.5%
	Route: Other	82.3%	50.9%
	Route: Unspecified	13.6%	42.7%

### Online presence/communication and partnership profile

We found that regenerative medicine/stem cell clinics operating in Tijuana had an active online presence, particularly through dedicated websites (94.5%, *n* = 104), Facebook accounts (65.5%, *n* = 72), Twitter accounts (26.4%, *n* = 29), and “other” forms of online presence using other websites/blogs/website applications (37.3%, *n* = 41). Of the websites detected, 85.5% (*n* = 94) of clinics in Tijuana had an embedded contact form for prospective clientele to inquire about services, 88.2% (*n* = 97) of clinics in Tijuana shared the public address of their location, and 12.7% (*n* = 14) also mentioned partners on their websites, including other clinics, institutions, or agencies. Eleven websites claimed to have partners within Mexico only, 2 websites claimed partners in countries outside of Mexico and the USA, and only 1 website claimed partners in both countries. Websites in Tijuana communicated in Spanish and English roughly equally (40%, *n* = 44), and 20.2% (*n* = 22) of websites utilized both languages.

### Tokens of scientific legitimacy

The majority of Tijuana websites did not mention any affiliation to academic universities or research centers, or other overt tokens of scientific legitimacy. We found that only one (0.9%) of the Tijuana stem cell clinic websites cited an academic affiliation (compared to San Diego clinics that had 11 claims of academic affiliation [7.5%]), though 13.6% (*n* = 15) cited an affiliation to professional societies and networks. Additionally, 6.4% (*n* = 7) of these websites included scientific-related self-publications, such as blog posts or self-authored opinion pieces referring to the efficacy of regenerative services offered. Peer-reviewed articles or other reputable forms of scientific information sources were observed on 6 websites (5.5%), however all of these articles were not relevant for clinical application of SCBIs as they cited to animal model experimental data as evidence. Another important observation of purported scientific and regulatory legitimacy were websites that claimed to be licensed for the practice of regenerative medicine (11.8%, *n* = 13) based on approval by regulatory agencies in Mexico, though only 10 clinics in total were licensed at the time of this study by these same authorities.

### Intervention details

A wide range of different regenerative medicine treatments were offered by Tijuana stem cell clinics, with an average of 8.15 treatment applications marketed for a variety of diverse health conditions. Tijuana stem cell clinics mentioned the use of unspecified cell types (26.4%, *n* = 29), embryonic stem cells (3.6%, *n* = 4), and sometimes did not mention the source of tissue used for marketed regenerative medicine treatments (32.7%, *n* = 36). The majority of websites (61.8%, *n* = 68) mentioned the use of autologous tissue, and 67.3% (*n* = 74) of websites also advertised PRP derived treatments (see Table 1). Regarding the routes of administration marketed, 42.7% (*n* = 47) of Tijuana websites did not specify the route of administration, while half (50.9%, *n* = 56) offered “other” routes of administration, which consisted of intraarticular, intradermal routes, and nine that mentioned intrathecal delivery.

We also assessed disclosure of potential health and safety risks by websites, including whether language about possible risks or adverse events associated with therapies were communicated. Generally, we considered disclosure of the risk of adverse event to fall into categories of “not mentioned,” “safe,” “minor,” “moderate,” and “severe.” Claims that the therapy was “safe” included when it was represented that therapy would not interfere with the daily activities of the patient. Minor adverse events were those that did not interfere with daily activities but required medication to resolve. A moderate adverse event was categorized as one that interfered with daily activities of the patient and required treatment as an outpatient. A severe adverse event was one that interfered with the daily activities of a patient and required medical treatment and hospitalization and could be considered life threatening. Based on these categorizations, risk disclosed on websites was most commonly “*not mentioned*” at all (55.4%, *n* = 61), followed by “*safe*” (38.2%, *n* = 42), and “*minor*” (6.0%, *n* = 7). No website mentioned the possibility for “*severe*” risk for any treatment offered.

Regarding information about possible treatment expectations for patients, the most common representation made by Tijuana clinic websites was that the prognosis for treatment was “*unclear*” (49.1%, *n* = 54), followed by “*improvement only*” (48.2%, *n* = 53), while “*both*” (improvement and cure) was mentioned only 2.7% (*n* = 3) of the time. In general, the expectations for marketed stem cell/regenerative medicine therapies among Tijuana clinics reviewed were labeled as “unspecified,” while for PRP they were mostly marketed as “improvement only.”

### Costs of services

We also captured the average costs for one treatment session for those websites that publicly advertised this information on their stem cell clinic websites. Importantly, the costs mentioned in this study do not reflect any pricing for transport, accommodations, or other services that may be bundled in some cases by Tijuana businesses offering these therapies. Our observed pricing data indicated that the average cost of stem cell/regenerative medicine treatments marketed in Tijuana for single sessions was $3691.76. However, this result was driven by two outliers in our Tijuana website group that greatly increased the apparent mean costs of treatments. Once we removed these outliers the average cost per treatment was $1550.67 for single sessions. We also observed a distinct price difference between websites offering PRP only services (lower) compared to sites that offered stem cell treatments only (higher), though there was not enough data to draw clear conclusions. There was also no specific and detailed pricing available that was stratified for different conditions treated, therapeutic routes, or tissue sources.

### San Diego clinics sub-analysis

**Fig. 2:**
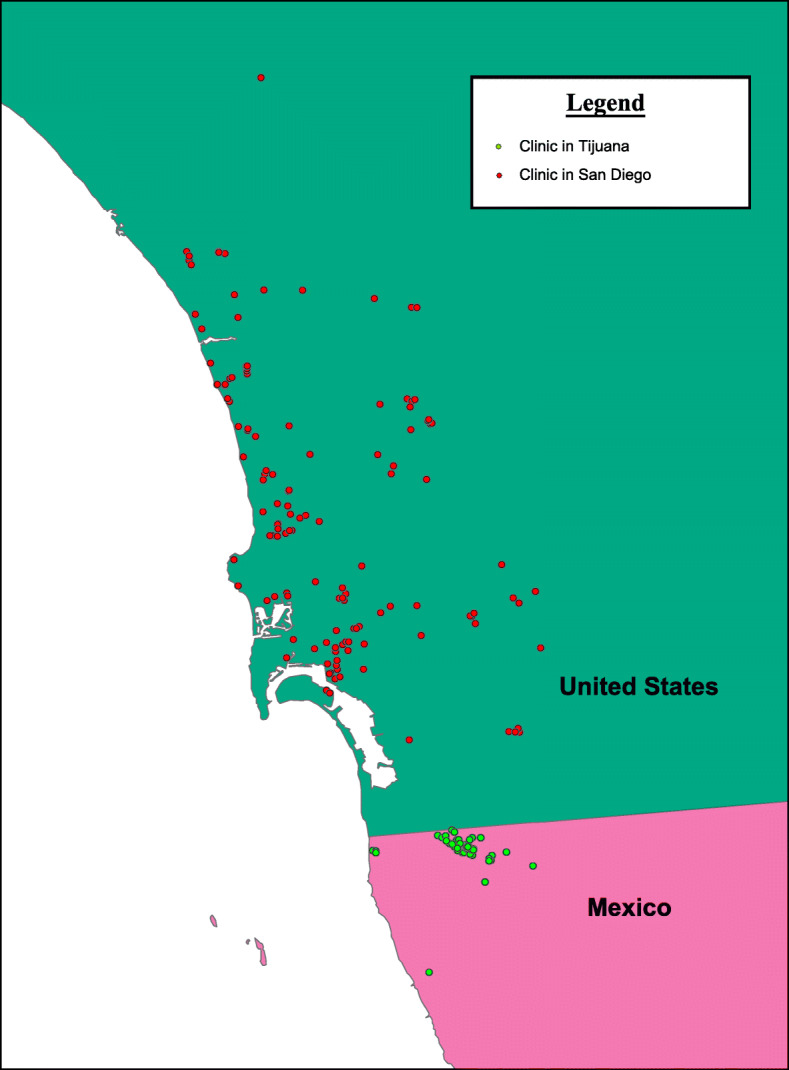
Map of San Diego and Tijuana regenerative medicine businesses/clinics reviewed in this study

Our sub-analysis of regenerative medicine websites marketing services online and located in San Diego (*n* = 147, see Fig. 2) found that these US-based businesses exhibited characteristics that were distinctly different from clinics detected in Tijuana, notably in the types of SCBI treatment options offered. There was a greater density of San Diego-based clinics found in our structured searches compared to clinics in Tijuana. These clinics were more likely to have a dedicated website or other forms of online presence, more likely to have contact forms, along with a greater number of claimed academic affiliations (7.5% vs 0.9%, *p* = 0.015). However, unlike Tijuana clinics, the vast majority of clinics only communicated online via English language. The language most commonly used was English alone for both San Diego and Tijuana websites, with San Diego having the greater proportion than Tijuana (95.2% and 40% respectively). For the actual interventions offered, San Diego clinics mentioned the use of autologous tissue more often than those located in Tijuana, and clinics notably had a higher proportion of websites advertising PRP derived treatments (87.1% vs 67.3%, *p* < 0.001). In comparison, Tijuana clinics offered a greater breadth of unproven SCBI treatment options, and though the average treatment costs were higher in Tijuana, a statistically significant difference was not observed.

## Discussion

A growing number of studies have examined the changing characteristics and continued globalization of the direct-to-consumer marketplace for regenerative medicine services. Many of these studies point to rapid growth in the number of new stem cell business websites, including in high-income countries like the United States, where clinics are exploiting gaps or loopholes in the regulatory framework [21–23]. While regulators have taken some action against unproven stem cell therapy providers (including the FDA seeking permanent injunctions to shut down stem cell companies), emerging and low-to-middle income markets—including Mexico, India, Thailand, and China—continue to act as key stem cell tourism destinations internationally due to their lack of sufficient oversight or enforcement of questionable SCBI providers [16, 22].

Many of the leading stem cell tourism destination countries are comparable in terms of socioeconomic development with Mexico, where an absence of direct regulation (e.g., no “Norma Official Mexicana” for SCBIs) has enabled the presence of suspect providers who market unproven SCBI services locally, to the USA, and abroad via the Internet. In this sense, Mexico exhibits similarities to other stem cell tourism countries, where low operating costs, policies prioritizing provider discretion, and lack of enforcement of any existing regulations, creates an environment for unregulated stem cell markets to thrive [8]. Further, the unique dynamic between Tijuana and San Diego due to close geographic proximity, cultural and social ties, and relative ease of crossing the border, may be giving rise to a form of cross-border stem cell tourism specific to this region that also includes competition and specialization.

To better explore this cross-border market, our study used structured web searches to identify and characterize 76 stem cell clinics located in Tijuana that also had a marketing presence online via direct-to-consumer-advertising. These online stem cell clinics predominantly used dedicated websites and social media platforms (including Facebook and Twitter) to extend the reach of their services to both English and Spanish language speaking audiences. Most of these websites advertised autologous tissue as the source of stem cell interventions, though others did not specifically mention the cell type used, and a smaller number marketed embryonic stem cell-based therapy. For comparison purposes, most of the clinics we reviewed located in San Diego only offered PRP treatment, in stark comparison to the higher number of clinics in Tijuana offering SCBIs or regenerative treatment options that may be promising, but remain largely unsubstantiated for clinical use and were not marketed in the context of participation in a clinical trial.

Though autologous use and minimally manipulated stem-cell derived treatments are generally considered safe in the US and Mexico, these websites nevertheless raise concerns about the exact types of therapies offered and the efficacy of these treatments for specific health conditions advertised. Furthermore, findings raise concerns about levels of transparency of services marketed on these websites direct-to-consumer, including issues regarding the quality and type of stem cell/blood products used, communication about potential risks and limited evidence of efficacy, and whether these clinics are subject to sufficient regulatory oversight to ensure adequate patient safety protection [24]. Specifically, the majority of websites we reviewed did not include any information about possible risks or adverse events of treatments offered similar to findings in other studies, though overt promises of positive treatment outcomes were also muted [20].

In Mexico, COFEPRIS governs all domestic licensing for regenerative medicine purposes. According to existing regulations, all establishments that deal with extracting, storing, preparing, transplanting, transfusing, or administering tissues or cells must be licensed. This is specified in Mexico’s General Health Act (GHA; under Title 14, Chapter 1, article 315), which serves as the legal framework that describes all norms and policies relevant to a person’s rights to the protection of their health in accordance with the Mexican constitution. However, there appears to be a discrepancy in our study results regarding the number of stem cell clinics we detected operating in Tijuana and those claiming licensure approval, which indicates there may be a number of businesses or clinics marketing these therapies online that are unlicensed by COFEPRIS.

COFEPRIS offers three distinct types of licenses, one that licenses businesses/clinics for the collection of stem cells, another one for biobanking of tissues including storage of stem cells, and one for regenerative medicine therapies. Subdireccion Ejecutiva de Autorizaciones en Servicios de Salud (SEASS), a subsection of COFEPRIS, publishes an official public list of businesses with approved licenses for regenerative medicine on the official government website Gobierno de México, which was used to cross-reference stem cell clinics reviewed during this study [25]. In total, 17 out of 52 licenses nationally (32.7%) were granted to Tijuana (the highest in the country), seven of which were licenses only for the collection or storage of stem cells. Our study detected 76 clinics in Tijuana, of which 13 claimed COFEPRIS licensing approval, representing 3 more providers than were licensed by COFEPRIS for SCBI’s. Upon further inspection, 6 of these clinics matched the COFEPRIS list for *either* business name or address, but only 1 matched accurately for *both* business name and address.

These results bring into question the legitimacy of claimed licensure approval by Tijuana clinics actively marketed online and whether there is sufficient oversight by COFEPRIS. In fact, COFEPRIS officials have been quoted in the past stating that one of the main oversight tools that should be utilized by the public is patients self-reporting to regulatory authorities any unauthorized clinics they become aware of. However, patients are generally not well versed in regulatory aspects concerning SCBIs nor do they have the tools to prosecute the legitimacy of claims regarding clinic licensure. This is in addition to the challenges already faced by patients in assessing the scientific legitimacy and purported evidence for efficacy of marketed treatments, all information points needed in order to fully ascertain the risks versus benefits of seeking and paying for these services. As such, patients are more likely to report a treatment-related complication ex-post facto.

Additionally, patients may engage in medical tourism specifically seeking access to experimental stem cell therapy options in countries with more lax regulation than in their home countries. This is not just an issue that implicates Mexico, as the promotion of the global stem cell tourism industry includes numerous low-to-middle income countries including India, Thailand, the Caribbean, Latin America, but is also emerging as a challenge for high-income markets such as the USA, Europe, Australia, and Japan [6, 16, 26]. Hence, coordinated action is required on several fronts, the first of which should focus on efforts to harmonize regulatory frameworks internationally while continuing to improve existing regulations for the approval of biologic and cellular therapies that have been proven safe and effective for clinical use. Further, better enforcement of existing licensure requirements, regulatory product standards, and laws against false and misleading online marketing will also be crucial to ensuring the integrity of stem cell therapy locally, abroad, and online and offline.

### Study limitations

Our study has certain limitations. Our sub-analysis of San Diego stem cell clinics found that San Diego had a higher number of websites compared to Tijuana (147 and 110 respectively), which is in line with previous studies that suggest the USA has the most websites for regenerative medicine therapies [15]. However, this difference could be driven by different factors such as population size of a particular region or country, or search engine marketing or optimization expenditures that differ between US and Mexican businesses, or alternatively, fewer locations in Tijuana due to limitations on infrastructure and personnel needed to operate clinics. For this reason, results are not generalizable and are only a snapshot of the characteristics of online marketing for stem cell clinics for a specific search setting aimed at identifying stem cell clinics in Tijuana and San Diego. The data regarding pricing reported in this study represents a small number of websites that included this data (17 in Tijuana, 30 in San Diego). The data in some cases represents some but not all of the marketed procedures by a particular website reviewed. Furthermore, it is difficult to categorize and compare pricing for specific procedures due to the fact that most websites reviewed did not offer full or transparent information regarding specific details that could affect pricing (i.e., need for hospitalization, route of administration, tissue source) and did not mention additional bundled costs which are common in the case of medical tourism (i.e., follow-up medical checkups, lodging and transportation costs). Future studies should focus on developing approaches to generate more robust SCBI pricing data for services marketed online, including potentially contacting vendors directly for additional information. Additionally, we could not analyze the specific marketing strategies or quantify the number of stem cell clinics in either Tijuana or San Diego that are not indexed or otherwise directly observable using our structured web searches, further limiting the generalizability of the stem cell clinic characteristics reviewed. Finally, this study’s findings are limited to the time period the study was conducted and may not be generalizable to overall trends and changes in online stem cell therapy marketing.

## Conclusions

Our study found that availability to Tijuana stem cell clinic services can extend beyond national borders via the Internet. Results build on prior studies evidencing the presence of an unregulated digital marketplace involving direct-to-consumer marketing of regenerative medicine services locally and globally. Better oversight and enforcement of online direct-to-consumer marketing claims by corresponding country regulatory authorities, Internet service providers, and professional associations and medical licensing boards, coupled with global cooperation to address proliferation of questionable regenerative medicine providers in an increasing virtual world is needed. Such action is critical in order to ensure that these sources of access do not delegitimatize the future promise of regenerative medicine and its application to improving human health.

## Supplementary Information

**Additional file 1: Supplementary Table 1.** Search terms used to find online marketing for regenerative medicine clinics in Tijuana and San Diego, with 148 concepts queried in both English and Spanish. **Supplementary Table 2.** List of variables collected from websites for regenerative medicine clinics in Tijuana and San Diego.

## Data Availability

Underlining data is available upon reasonable request to author.

## Abbreviations

COFEPRIS: Comisión Federal para la Protección contra Riesgos Sanitarios
FDA: US Food and Drug Administration
HCT/P: Human cells, tissues and cellular and tissue-based products
GHA: Mexico’s General Health Act
NOM: Norma Official Mexicana
PHS: Public Health Services Act
PRP: Platelet-rich plasma
SEASS: Subdireccion Ejecutiva de Autorizaciones en Servicios de Salud
SCBI: Stem cell-based interventions
