# Supplementary material for: Online marketing practices of regenerative medicine clinics in US-Mexico border region: a web surveillance study
Source: Stem Cell Res Ther. 2021 Mar 18;12:189. doi: 10.1186/s13287-021-02254-4 (PMC7977255; doi:10.1186/s13287-021-02254-4)
Supplement: Supplementary file 1 — Additional file 1: Supplementary Table 1. Search terms used to find online marketing for regenerative medicine clinics in Tijuana and San Diego, with 148 concepts queried in both English and Spanish. Supplementary Table 2. List of variables collected from websites for regenerative medicine clinics in Tijuana and San Diego. [file 13287_2021_2254_MOESM1_ESM.docx]

**APPENDIX**

**Supplementary Table 1.** Search terms used to find online marketing for regenerative medicine clinics in Tijuana and San Diego, with 148 concepts queried in both English and Spanish

| 1. Stem cell regenerative medicine San Diego 2. Células madre medicina regenerativa San Diego | 1. Stem cell regenerative medicine Tijuana 2. Células madre medicina regenerativa Tijuana |
| --- | --- |
| 1. Stem cell clinic Tijuana 2. Clínica de células madre Tijuana | 1. Stem cell therapy Tijuana 2. Terapia con células madre Tijuana |
| 1. Stem cell treatment Tijuana 2. Tratamiento con células madre Tijuana | 1. Stem cell treatment San Diego 2. Tratamiento con células madre San Diego |
| 1. Stem cell clinic San Diego 2. Clínica de células madre San Diego | 1. Stem cell therapy San Diego 2. Terapia con células madre San Diego |
| 1. Regenerative medicine clinic Tijuana 2. Clínica de medicina regenerativa Tijuana | 1. Regenerative medicine therapy Tijuana 2. Terapia de medicina regenerativa Tijuana |
| 1. Regenerative medicine clinic San Diego 2. Clínica de medicina regenerativa San Diego | 1. Regenerative medicine therapy San Diego 2. Terapia de medicina regenerativa San Diego |
| 1. Regenerative medicine treatment Tijuana 2. Tratamiento con medicina regenerativa Tijuana | 1. Regenerative medicine treatment San Diego 2. Tratamiento con medicina regenerativa San Diego |
| 1. Stem cell doctor Tijuana 2. Doctor en células madre Tijuana | 1. Stem cell doctor San Diego 2. Doctor en células madre San Diego |
| 1. Regenerative medicine doctor Tijuana 2. Doctor medicina regenerativa Tijuana | 1. Regenerative medicine doctor San Diego 2. Doctor medicina regenerativa San Diego |
| 1. San Diego stem cell center 2. Centro de células madre San Diego | 1. Stem cell injections San Diego 2. Inyecciones de células madre San Diego |
| 1. San Diego regenerative medicine center 2. San Diego centro de medicina regenerativa | 1. Adipose stem cell therapy San Diego 2. Terapia con células madre adiposas |
| 1. Tijuana stem cell center 2. Centro de células madre Tijuana | 1. Adipose stem cell treatment San Diego 2. Tratamiento con células madre adiposas San Diego |
| 1. Tijuana regenerative medicine center 2. Tijuana Centro de medicina regenerativa | 1. Stromal Vascular Fraction stem cell therapy San Diego 2. Terapia con Fracción vascular estromal células madre San Diego |
| 1. Stem cell injections Tijuana 2. Inyecciones de células madre Tijuana | 1. Stromal Vascular Fraction stem cell treatment San Diego 2. Tratamiento con Fracción vascular estromal células madre San Diego |
| 1. Adipose stem cell therapy Tijuana 2. Terapia con células madre adiposas Tijuana | 1. Embryonic stem cell treatment San Diego 2. Tratamiento con células madre embrionarias San Diego |
| 1. Adipose stem cell treatment Tijuana 2. Tratamiento con células madre adiposas Tijuana | 1. Embryonic stem cell therapy San Diego 2. Terapia con células madre embrionarias San Diego |
| 1. Stromal Vascular Fraction stem cell therapy Tijuana 2. Terapia con Fracción vascular estromal células madre Tijuana | 1. Amniotic stem cell treatment San Diego 2. Tratamiento con células madre amnióticas San Diego |
| 1. Stromal Vascular Fraction stem cell treatment Tijuana 2. Tratamiento con Fracción vascular estromal células madre Tijuana | 1. Amniotic stem cell therapy San Diego 2. Terapia con células madre amnióticas San Diego |
| 1. Embryonic stem cell treatment Tijuana 2. Tratamiento con células madre embrionarias Tijuana | 1. Fat-derived stem cell treatment San Diego 2. Tratamiento con células madre derivadas de adipocitos San Diego |
| 1. Embryonic stem cell therapy Tijuana 2. Terapia con células madre embrionarias Tijuana | 1. Fat-derived stem cell therapy San Diego 2. Terapia con células madre derivadas de adipocitos San Diego |
| 1. Amniotic stem cell treatment Tijuana 2. Tratamiento con células madre amnióticas Tijuana | 1. Fat-derived stem cell treatment Tijuana 2. Tratamiento con células madre derivadas de adipocitos Tijuana |
| 1. Amniotic stem cell therapy Tijuana 2. Terapia con células madre amnióticas Tijuana | 1. Fat-derived stem cell therapy Tijuana 2. Terapia con células madre derivadas de adipocitos Tijuana |
| 1. iPSC treatment Tijuana 2. Tratamiento con células madre pluripotentes inducidas Tijuana | 1. iPSC treatment San Diego 2. Tratamiento con células madre pluripotentes inducidas San Diego |
| 1. iPSC therapy Tijuana 2. Terapia con células madre pluripotentes inducidas Tijuana | 1. iPSC therapy San Diego 2. Terapia con células madre pluripotentes inducidas San Diego |
| 1. Bone marrow stem cell therapy Tijuana 2. Terapia con células madre de medula ósea Tijuana | 1. Bone marrow stem cell therapy San Diego 2. Terapia con células madre de medula ósea San Diego |
| 1. Bone marrow stem cell treatment Tijuana 2. Tratamiento con células madre de medula ósea Tijuana | 1. Bone marrow stem cell treatment San Diego 2. Tratamiento con células madre de medula ósea San Diego |
| 1. Adult stem cell therapy Tijuana 2. Terapia con células madre adultas Tijuana | 1. Adult stem cell therapy San Diego 2. Terapia con células madre adultas San Diego |
| 1. Adult stem cell treatment Tijuana 2. Tratamiento con células madre adultas Tijuana | 1. Adult stem cell treatment San Diego 2. Tratamiento con células madre adultas San Diego |
| 1. Pain stem cell therapy San Diego 2. Terapia con células madre para dolor San Diego | 1. Pain stem cell therapy Tijuana 2. Terapia con células madre para dolor Tijuana |
| 1. Pain stem cell clinic San Diego 2. Clínica de células madre para dolor San Diego | 1. Pain stem cell clinic Tijuana 2. Clínica de células madre para dolor Tijuana |
| 1. Pain stem cell treatment Tijuana 2. Tratamiento con células madre para dolor Tijuana | 1. Pain stem cell treatment San Diego 2. Tratamiento con células madre para dolor San Diego |
| 1. Pain stem cell center Tijuana 2. Centro de células madre para dolor Tijuana | 1. Pain stem cell center San Diego 2. Centro de células madre para dolor San Diego |
| 1. Sports injury stem cell therapy Tijuana 2. Terapia con células madre para lesiones deportivas Tijuana | 1. Sports injury stem cell therapy San Diego 2. Terapia con células madre para lesiones deportivas San Diego |
| 1. Sports injury stem cell treatment Tijuana 2. Tratamiento con células madre para lesiones deportivas Tijuana | 1. Sports injury stem cell treatment San Diego 2. Tratamiento con células madre para lesiones deportivas San Diego |
| 1. Sports injury stem cell clinic Tijuana 2. Clínica de células madre para lesiones deportivas Tijuana | 1. Sports injury stem cell clinic San Diego 2. Clínica de células madre para lesiones deportivas San Diego |
| 1. Sports injury stem cell center Tijuana 2. Centro de células madre para lesiones deportivas Tijuana | 1. Sports injury stem cell center San Diego 2. Centro de células madre para lesiones deportivas San Diego |
| 1. Arthritis stem cell treatment Tijuana 2. Tratamiento con células madre para artritis Tijuana | 1. Arthritis stem cell treatment San Diego 2. Tratamiento con células madre para artritis San Diego |
| 1. Arthritis stem cell clinic Tijuana 2. Clínica de células madre para artritis Tijuana | 1. Arthritis stem cell clinic San Diego 2. Clínica de células madre para artritis San Diego |
| 1. Arthritis stem cell therapy Tijuana 2. Terapia con células madre para artritis Tijuana | 1. Arthritis stem cell therapy San Diego 2. Terapia con células madre para artritis San Diego |
| 1. Arthritis stem cell center Tijuana 2. Centro de células madre para artritis Tijuana | 1. Arthritis stem cell center San Diego 2. Centro de células madre para artritis San Diego |
| 1. Anti-aging stem cell therapy Tijuana 2. Terapia anti-envejecimiento con células madre Tijuana | 1. Anti-aging stem cell therapy San Diego 2. Terapia anti-envejecimiento con células madre San Diego |
| 1. Anti-aging stem cell treatment Tijuana 2. Tratamiento anti-envejecimiento con células madre Tijuana | 1. Anti-aging stem cell treatment San Diego 2. Tratamiento anti-envejecimiento con células madre San Diego |
| 1. Anti-aging stem cell clinic Tijuana 2. Clínica de células madre anti-envejecimiento Tijuana | 1. Anti-aging stem cell clinic San Diego 2. Clínica de células madre anti-envejecimiento San Diego |
| 1. Anti-aging stem cell center Tijuana 2. Centro de células madre anti-envejecimiento Tijuana | 1. Anti-aging stem cell center San Diego 2. Centro de células madre anti-envejecimiento San Diego |
| 1. Cosmetic stem cell treatment Tijuana 2. Tratamiento cosmético con células madre Tijuana | 1. Cosmetic stem cell treatment San Diego 2. Tratamiento cosmético con células madre San Diego |
| 1. Cosmetic stem cell therapy Tijuana 2. Terapia cosmética con células madre Tijuana | 1. Cosmetic stem cell therapy San Diego 2. Terapia cosmética con células madre San Diego |
| 1. Cosmetic stem cell clinic Tijuana 2. Clínica cosmética de células madre Tijuana | 1. Cosmetic stem cell clinic San Diego 2. Clínica cosmética de células madre San Diego |
| 1. Cosmetic stem cell center Tijuana 2. Centro cosmético de células madre Tijuana | 1. Cosmetic stem cell center San Diego 2. Centro cosmético de células madre San Diego |
| 1. Diabetes stem cell treatment Tijuana 2. Tratamiento con células madre para diabetes Tijuana | 1. Diabetes stem cell treatment San Diego 2. Tratamiento con células madre para diabetes San Diego |
| 1. Diabetes stem cell therapy Tijuana 2. Terapia con células madre para diabetes Tijuana | 1. Diabetes stem cell therapy San Diego 2. Terapia con células madre para diabetes San Diego |
| 1. Diabetes stem cell clinic Tijuana 2. Clínica de células madre para diabetes Tijuana | 1. Diabetes stem cell clinic San Diego 2. Clínica de células madre para diabetes San Diego |
| 1. Diabetes stem cell center Tijuana 2. Centro de células madre para diabetes Tijuana | 1. Diabetes stem cell center San Diego 2. Centro de células madre para diabetes San Diego |
| 1. Heart disease stem cell treatment Tijuana 2. Tratamiento con células madre para enfermedad cardiaca Tijuana | 1. Heart disease stem cell treatment San Diego 2. Tratamiento con células madre para enfermedad cardiaca San Diego |
| 1. Heart disease stem cell therapy Tijuana 2. Terapia con células madre para enfermedad cardiaca Tijuana | 1. Heart disease stem cell therapy San Diego 2. Terapia con células madre para enfermedad cardiaca San Diego |
| 1. Heart disease stem cell clinic Tijuana 2. Clínica de células madre para enfermedad cardiaca Tijuana | 1. Heart disease stem cell clinic San Diego 2. Clínica de células madre para enfermedad cardiaca San Diego |
| 1. Heart disease stem cell center Tijuana 2. Centro de células madre para enfermedad cardiaca Tijuana | 1. Heart disease stem cell center San Diego 2. Centro de células madre para enfermedad cardiaca San Diego |
| 1. Spinal cord injury stem cell therapy Tijuana 2. Terapia con células madre para lesiones de medula espinal Tijuana | 1. Spinal cord injury stem cell therapy San Diego 2. Terapia con células madre para lesiones de medula espinal San Diego |
| 1. Spinal cord injury stem cell treatment Tijuana 2. Tratamiento con células madre para lesiones de medula espinal Tijuana | 1. Spinal cord injury stem cell treatment San Diego 2. Tratamiento con células madre para lesiones de medula espinal San Diego |
| 1. Spinal cord injury stem cell clinic Tijuana 2. Clínica de células madre para lesiones de medula espinal Tijuana | 1. Spinal cord injury stem cell clinic San Diego 2. Clínica de células madre para lesiones de medula espinal San Diego |
| 1. Spinal cord injury stem cell center Tijuana 2. Centro de células madre para lesiones de medula espinal Tijuana | 1. Spinal cord injury stem cell center San Diego 2. Centro de células madre para lesiones de medula espinal San Diego |
| 1. Stroke stem cell treatment Tijuana 2. Tratamiento con células madre para infarto cerebral Tijuana | 1. Stroke stem cell treatment San Diego 2. Tratamiento con células madre para derrame cerebral San Diego |
| 1. Stroke stem cell therapy Tijuana 2. Terapia con células madre para infarto cerebral Tijuana | 1. Stroke stem cell therapy San Diego 2. Terapia con células madre para infarto cerebral San Diego |
| 1. Stroke stem cell clinic Tijuana 2. Clínica de células madre para infarto cerebral Tijuana | 1. Stroke stem cell clinic San Diego 2. Clínica de células madre para infarto cerebral San Diego |
| 1. Stroke stem cell center Tijuana 2. Centro de células madre para infarto cerebral Tijuana | 1. Stroke stem cell center San Diego 2. Centro de células madre para infarto cerebral San Diego |
| 1. Multiple sclerosis stem cell therapy Tijuana 2. Terapia con células madre para esclerosis múltiple Tijuana | 1. Multiple sclerosis stem cell therapy San Diego 2. Terapia con células madre para esclerosis múltiple San Diego |
| 1. Multiple sclerosis stem cell treatment Tijuana 2. Tratamiento con células madre para esclerosis múltiple Tijuana | 1. Multiple sclerosis stem cell treatment San Diego 2. Tratamiento con células madre para esclerosis múltiple San Diego |
| 1. Multiple sclerosis stem cell clinic Tijuana 2. Clínica de células madre para esclerosis múltiple Tijuana | 1. Multiple sclerosis stem cell clinic San Diego 2. Clínica de células madre para esclerosis múltiple San Diego |
| 1. Multiple sclerosis stem cell center Tijuana 2. Centro de células madre para esclerosis múltiple Tijuana | 1. Multiple sclerosis stem cell center San Diego 2. Centro de células madre para esclerosis múltiple San Diego |
| 1. Paralysis stem cell treatment Tijuana 2. Tratamiento con células madre para parálisis Tijuana | 1. Paralysis stem cell treatment San Diego 2. Tratamiento con células madre para parálisis San Diego |
| 1. Paralysis stem cell therapy Tijuana 2. Terapia con células madre para parálisis Tijuana | 1. Paralysis stem cell therapy San Diego 2. Terapia con células madre para parálisis San Diego |
| 1. Paralysis stem cell clinic Tijuana 2. Clínica de células madre para parálisis Tijuana | 1. Paralysis stem cell clinic San Diego 2. Clínica de células madre para parálisis San Diego |
| 1. Paralysis stem cell center Tijuana 2. Centro de células madre para parálisis Tijuana | 1. Paralysis stem cell center San Diego 2. Centro de células madre para parálisis San Diego |
| 1. PRP treatment Tijuana 2. Tratamiento con Plasma rico en plaquetas Tijuana | 1. PRP treatment San Diego 2. Tratamiento con Plasma rico en plaquetas San Diego |
| 1. PRP therapy Tijuana 2. Terapia con Plasma rico en plaquetas Tijuana | 1. PRP therapy San Diego 2. Terapia con Plasma rico en plaquetas San Diego |

**Supplementary Table 2.** List of variables collected from websites for regenerative medicine clinics in Tijuana and San Diego.

| **Category** | **Variable Description** |
| --- | --- |
| Online communication and partnership profile | Does the clinic have a Facebook page link on its website? |
|  | Does the clinic have a Twitter page link on its website? |
|  | Does the clinic have other forms of social media or online presence? |
|  | Languages used on the website |
|  | Telephone number provided |
|  | E-mail provided |
|  | Contact form provided within website |
|  | Mailing address provided |
|  | Does the website state more than one location in San Diego or Tijuana? |
|  | Links to other clinics or suppliers through website or social media |
|  | Partners mentioned |
|  | Location of partners |
| Claims to scientific legitimacy | Does the clinic claim academic affiliation? |
|  | Does the clinic claim affiliation to a hospital or medical center? |
|  | Does the clinic claim affiliation to a professional society or network? |
|  | Does the clinic claim affiliation with another authority? |
|  | Does the clinic claim to be licensed for regenerative medicine? |
|  | Website claims any FDA approval |
|  | Website claims any COFEPRIS approval |
|  | Website claims accreditation/certification from a professional organization |
|  | Does the clinic claim to be conducting approved clinical trials? |
|  | Claims approved patent |
|  | Claims patent is pending |
|  | Evidence cited (including company statements, patient testimonials, etc.) |
|  | Peer-reviewed evidence cited |
|  | Publications of in lay press, or blogs cited to support claims |
|  | Clinic publications on website (including blog posts) |
| Intervention details | Cost per each session |
|  | Number of different diseases treated |
|  | Diseases treated |
|  | Outcome expectation |
|  | Risk communication |
|  | Country from where the tissue product originates |
|  | Marketed cell type unspecified |
|  | Adult stem cell marketed |
|  | Embryonic stem cell marketed |
|  | Induced pluripotent stem cell marketed |
|  | Umbilical cord blood, placental or amniotic fluid stem cells marketed |
|  | Tissue Source |
|  | Route of administration unspecified |
|  | PO route of administration mentioned? |
|  | Topical route of administration mentioned? |
|  | IV route of administration mentioned? |
|  | Intrathecal route of administration mentioned? |
|  | IM route of administration mentioned? |
|  | Route of administration other than above mentioned? |
|  | Does the clinic offer PRP therapies? |
|  | Stated number of cases treated |
|  | Does the clinic mention follow-up to stem cell therapies offered? |
